# FPGA Realizations of Chaotic Epidemic and Disease Models Including Covid-19

**DOI:** 10.1109/ACCESS.2021.3055374

**Published:** 2021-01-28

**Authors:** M. Elnawawy, F. Aloul, A. Sagahyroon, A. S. Elwakil, Wafaa S. Sayed, Lobna A. Said, S. M. Mohamed, Ahmed G. Radwan

**Affiliations:** 1 Department of Computer Science and EngineeringAmerican University of Sharjah 47767 Sharjah 26666 United Arab Emirates; 2 Electrical and Computer EngineeringUniversity of Sharjah 59105 Sharjah 27272 United Arab Emirates; 3 Department of Electrical and Computer EngineeringUniversity of Calgary 2129 Calgary AB T2N 1N4 Canada; 4 Nanoelectronics Integrated Systems Center (NISC)Nile University 120639 Giza 16453 Egypt; 5 Engineering Mathematics and Physics DepartmentFaculty of EngineeringCairo University 63526 Giza 12613 Egypt; 6 School of Engineering and Applied SciencesNile University 120639 Giza 12588 Egypt

**Keywords:** Chaos, chaotic circuits, epidemic models, FPGA implementations

## Abstract

The spread of epidemics and diseases is known to exhibit chaotic dynamics; a fact confirmed by many developed mathematical models. However, to the best of our knowledge, no attempt to realize any of these chaotic models in analog or digital electronic form has been reported in the literature. In this work, we report on the efficient FPGA implementations of three different virus spreading models and one disease progress model. In particular, the Ebola, Influenza, and COVID-19 virus spreading models in addition to a Cancer disease progress model are first numerically analyzed for parameter sensitivity via bifurcation diagrams. Subsequently and despite the large number of parameters and large number of multiplication (or division) operations, these models are efficiently implemented on FPGA platforms using fixed-point architectures. Detailed FPGA design process, hardware architecture and timing analysis are provided for three of the studied models (Ebola, Influenza, and Cancer) on an Altera Cyclone IV EP4CE115F29C7 FPGA chip. All models are also implemented on a high performance Xilinx Artix-7 XC7A100TCSG324 FPGA for comparison of the needed hardware resources. Experimental results showing real-time control of the chaotic dynamics are presented.

## I. Introduction

Deterministic chaos is a common behavior in continuous-time dynamical systems of differential equations with nonlinear terms, which exhibit aperiodicity, ergodicity and sensitivity to initial conditions [1]. These properties of chaotic systems are needed in many applications such as modeling of robots [2], motion control [3], Random Number Generation and encryption applications [4]. This demand on chaotic systems in various applications has encouraged the exploration of different methods for their analog and digital hardware realizations [5]–[7]. Meanwhile, mathematical models of biological systems have been associated with chaotic behavior long before the emergence of chaos theory and dates back to the logistic equation model of population growth [8]. Epidemics and infectious diseases modeling is a relatively difficult problem, since their dynamics vary largely from one outbreak to another. For such emerging and reemerging diseases, the causes and transfer processes are often poorly known and understood. Susceptible, infected, recovered and possibly also exposed classes of a given population need to be considered. Many models have been developed for viral infectious diseases such as Influenza and Ebola [9]–[14] and nearly all of them show chaotic dynamics even after vaccination is administrated [15]. The emergence of chaotic behavior can be attributed mostly to predator-prey and competition dynamics [16]–[18] as well as nonlinear interactions between cell populations such as in cancer models [19]–[22] and Parkinson’s disease [23], [24].

Recently, following the COVID-19 epidemic crisis, more attention has been directed towards epidemics and infectious diseases modeling and the study of their state behavior against different factors [25]. These models usually contain many parameters and are sensitive to variations in their values. Therefore the implementation of these model should be attempted with caution. In this regards it is known that fixed-point implementation of chaotic models is generally more reliable and reproducible than floating-point ones [26], [27]. In fixed-precision fixed-point addition, there is no mantissa alignment and hence, no rounding errors in addition operations. A fixed-point representation was used in [27] to implement modified versions of the product integration rules; which are used to solve differential equations. Moreover, fixed-point operations can be performed efficiently in any Hardware Description Language (HDL) and realized on FPGA modules providing the advantages of re-programmability, reduced hardware cost, high speed, noise immunity and reliability. Many chaotic systems have already been digitally realized and tested on FPGA platforms [28]–[30] and also using Field Programmable Analog Arrays (FPAAs) [31]. One major advantage of FPGA realizations is that they facilitate experimenting with various chaos control schemes and real-time chaotic time-series prediction algorithms [32].

In this work we show the feasibility of implementing complex chaotic epidemic models using fixed-point architectures on FPGAs on two different FPGA platforms to explore the utilization of resources. We present the realization process of three virus spreading models and one disease progress model. Sensitivity of the chaotic dynamics to parameter variation is explored through continuous bifurcation diagrams prior to implementation. Difficulty arises from several aspects: (i) all the considered models have many parameters (17 parameters in the case of the Ebola model for example) and (ii) the sensitivity of chaotic behavior with respect to all these parameters needs to be carefully investigated before implementation. (iii) All models require several multiplication operations (7 multiplications and 1 division in the Cancer model for example). While it is possible for chaotic systems implemented on FPGAs for the purposes of encryption applications to be deigned such that they contain very few parameters and a limited number of multiplication/division operations [29], this is clearly not the case in epidemic models. It is worth noting that an analog circuit realization of a tumor growth model was recently presented in [33]. To the best of our knowledge, no FPGA realization of an epidemic or disease model on an FPGA platform has yet been reported in the literature. This article shows the feasibility of implementing these complex models efficiently on FPGAs for real-time simulation, control and prediction purposes.

This article is organized as follows: section II presents the investigated models and explores their chaotic dynamics. Section III presents the first proposed FPGA design procedure based on an Altera Cyclone IV EP4CE115F29C7 FPGA chip. A second implementation based on a Xilinx Artix-7 XC7A100TCSG324 chip is also reported. Section IV shows the FPGA resource utilization facts and experimental results.

## II. Selected Models

In this section, we describe the four models that are considered in this work. The details of these models and their respective derivation assumptions can be found in [11], [13], [25] and [34].

### A. Ebola Model

In [13], a 4-D model for the spread of the Ebola virus in West Africa between 2013 and 2016 was proposed. This deterministic model suggests that societal and environmental conditions were conducive to the propagation of Ebola. Once the epidemic had broken out, its propagation was driven by a few predominant processes. The spread model based on recorded time series data of $x_{1}$ and its higher order derivatives $y_{1,2,3}$ optimally fit the following model

\begin{align*} \dot {x}_{1}=&a_{1}y_{1}y_{3}+a_{2}y_{1}^{2}-a_{3}x_{1}y_{1} \\ \dot {y}_{2}=&y_{3},\dot {y}_{1}=y_{2} \\ \dot {y}_{3}=&f(x_{1},y_{1},y_{2},y_{3}) \tag{1}\end{align*}

where $f(\cdot)$ is a nonlinear function with 14 terms given by

\begin{align*} f=&b_{1}+b_{2}y_{3}+b_{3}y_{3}^{3}-b_{4}y_{2}-b_{5}y_{2}^{2}+b_{6}y_{1}-b_{7}y_{1}y_{3} \\&+\,b_{8}y_{1}y_{2}-b_{9}y_{1}^{2}-b_{10}x_{1}-b_{11}x_{1}y_{3}-b_{12}x_{1}y_{2} \\&+\,b_{13}x_{1}y_{1}+b_{14}x_{1}^{2} \tag{2}\end{align*}

**FIGURE 1.**
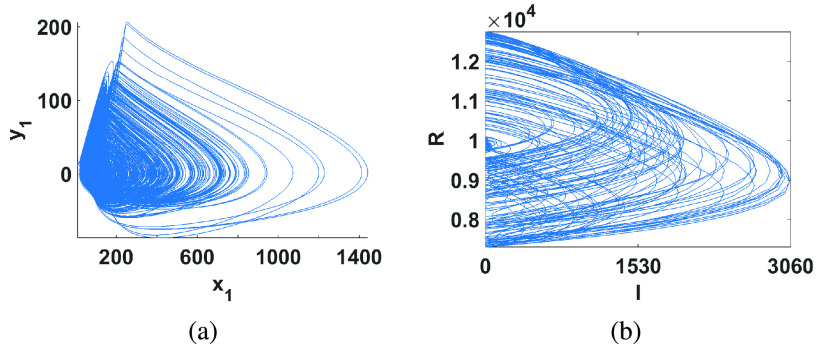
Numerical simulations of the discretized (a) Ebola model with initial conditions $x_{1}(0)=y_{1}(0)=10,y_{2}(0)=y_{3}(0)=0$ showing projection in the $x_{1}-y_{1}$ plane and (b) Influenza model with initial conditions $I(0)=10$ and $R(0)=8500$ projected in the $I-R$ plane (the total number of birds ($R+I$) is 10,000).

This model can be easily discretized using an Euler method with a suitable step size $h$. Figure 1(a) shows the simulation results of the discretized system with $h=10^{-4}$ and for the parameter values $(a_{1},a_{2},a_{3})=(10^{-4},1.5,1),\,\,(b_{1},b_{2},\ldots.b_{14})=(5800,3.8,2\times 10^{-5},1900,0.15,35\times 10^{3},0.05,15,1100,18\times 10^{3},0.07,25,300,180)$. Prior to considering an implementation of this system, the type of response obtained at different values of the parameters and the sensitivity to parameter variations needs to be studied. This was done by considering variations in narrow steps of one parameter at a time while fixing the other parameters using bifurcation diagrams. A bifurcation diagram reveals periodic windows and examines the robustness of the chaotic behavior versus parameter variations. For this system, the bifurcation diagram versus a chosen parameter was generated through plotting the value of the state $x_{1}$, every time it reaches a local maximum after discarding two thirds of the total number of points. Following extensive simulations of the system (2), we found that some parameters correspond to chaotic behavior only in very narrow ranges of their values, such as $b_{3}$. Other parameters can generate chaos for wider ranges such as $b_{6}$. In addition, some parameter variations drift the model into quasi-periodic state (donut-shaped attractor) such as when $a_{2}>1.5$ and $a_{3} < 1$.

### B. Influenza Model

The influenza model considered here was proposed in [11] for avian influenza in a seabird colony and is given by

\begin{align*} \dot {I}=&p(t)-\beta (I+R)I \\ \dot {R}=&\alpha I-\omega R \\ p(t)=&p(1-p_{1}\sin 2\pi t) \tag{3}\end{align*}

**FIGURE 2.**
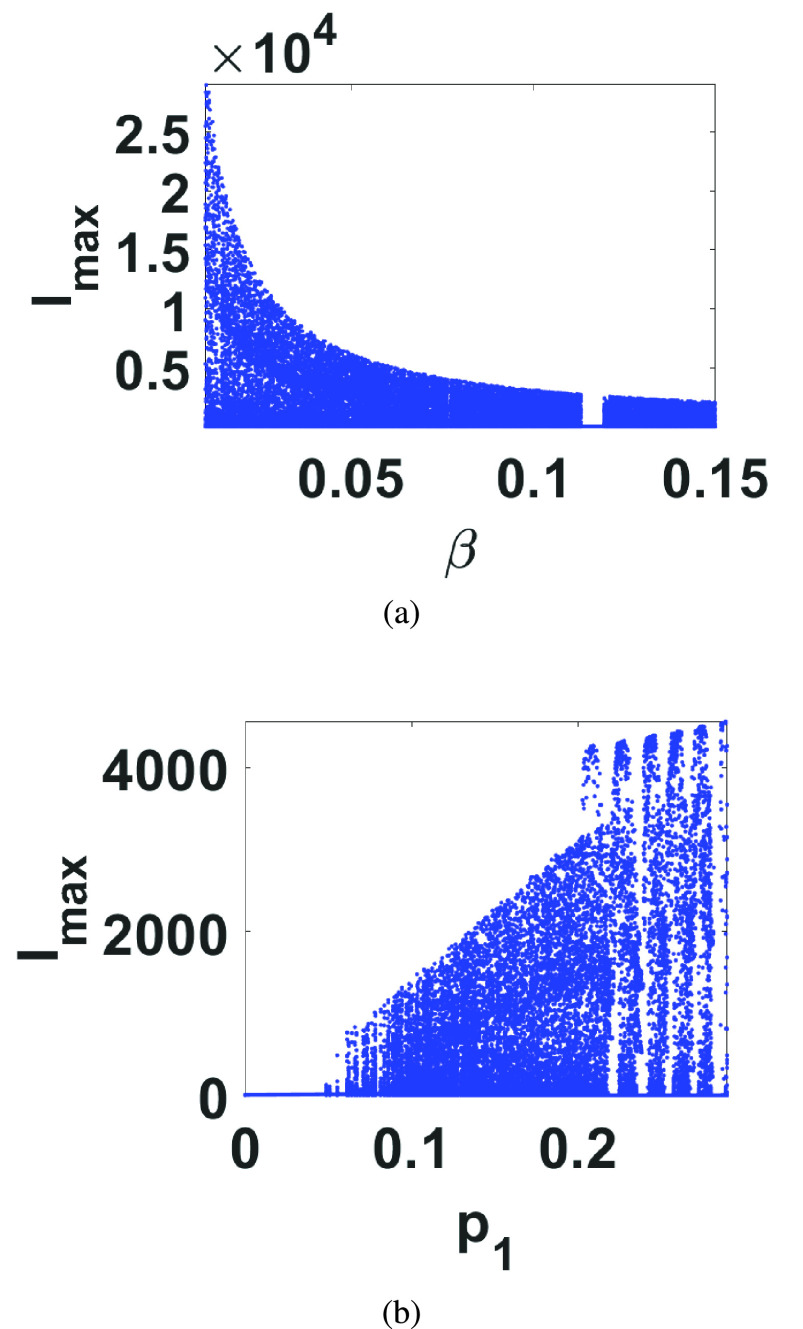
Bifurcation diagrams of the discretized Influenza model against (a) $\beta $ and (b) $p_{1}$ with the same initial conditions as in Fig. 1(b).

This model is obviously a non-autonomous periodically forced model where $I$ and $R$ respectively correspond to the infected and recovered individuals. $p$ may be interpreted as the recruitment rate of infectious individuals, $\beta $ is the transmission rate constant, $\alpha $ is the recovery rate constant and $\omega $ is the natural death rate constant. The driving forcing function $p(t)$ oscillates with a period of 1 year corresponding to the annual breeding season. It is also straight forward to discretize this model using Euler’s backward method and the simulation results in this case are shown in Fig. 1(b) for $(\beta,\alpha,\omega,p,p_{1})=(0.1,100,0.05,10^{3},0.2)$ and using a step size $h=10^{-3}$. The effect of the parameter variations on the state variables in this system was also carefully examined using bifurcation diagrams. The best two parameters that result in the widest range of chaotic behavior were found to be $\beta $ and $p_{1}$ and the corresponding bifurcation diagrams are plotted in Fig. 2 versus $I_{max}$.

### C. COVID-19 Model

A recent model of the COVID-19 pandemic was proposed in [25] taking into consideration data from China, Japan, South Korea and Italy. This model is given by

**FIGURE 3.**
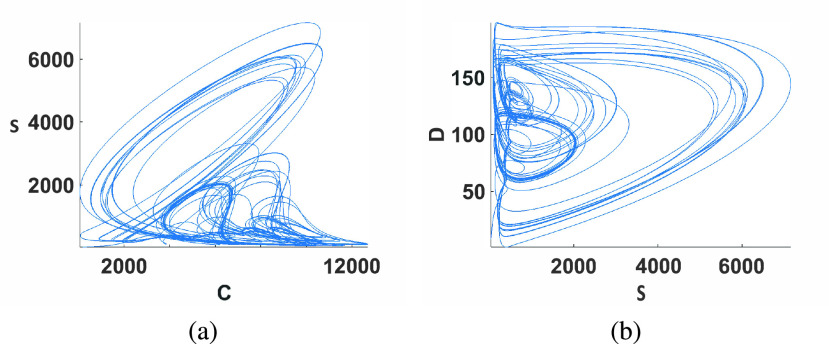
Numerical simulations of the discretized COVID-19 model with initial conditions $C(0)=184, S(0)=30,D(0)=8$ and discretization step $h=0.01$ (a) projection in the $C-S$ plane and (b) projection in the $S-D$ plane.

\begin{align*} \dot {C}=&a_{1}D-a_{2}D^{2}+a_{3}DS-a_{4}C+a_{5}CD-a_{6}CS \\ \dot {s}=&a_{7}DS-a_{8}CS \\ \dot {D}=&a_{9}D-a_{10}CD-a_{11}CS+a_{12}C^{2} \tag{4}\end{align*}

where $C (t)$ is the daily number of new cases, $S(t)$ is the daily additional severe cases and $D(t)$ is the daily number of new deaths. The model contains three multiplier-type nonlinearities ($DS, CD, CS$) in addition to two quadratic terms. Simulation results of this model after being discretized with an Euler method are shown in Fig. 3 with $(a_{1},a_{2},a_{3},a_{4},a_{5},a_{6},a_{7},a_{8},a_{9},a_{10},a_{11},a_{12})=\,\,(66,1.6966,0.148,0.8763,0.022843,0.0017342,0.05507,\,\,0.0008238,0.31303,0.0001057,1.008\times 10^{-5},1.734\times 10^{-6}$). For system (4), all parameters correspond to chaotic behavior only for narrow ranges or specific values. Hence, the solution exhibits extra sensitivity to the parameters values and extra care with the FPGA implementation is needed.

### D. Cancer Model

Many models of cancer growth are available in the literature from which we selected the 3-D model introduced in [34] and given by

\begin{align*} \dot {x}_{1}=&x_{1}(1-x_{1})-a_{1}x_{1}x_{2}-a_{2}x_{1}x_{3} \\ \dot {x}_{2}=&r_{1}x_{2}(1-x_{2})-a_{3}x_{1}x_{2} \\ \dot {x}_{3}=&\frac {r_{2}x_{1}x_{3}}{x_{1}+1}-a_{4}x_{1}x_{3}-a_{5}x_{3} \tag{5}\end{align*}

where $x_{1}$ is the number of tumor cells, $x_{2}$ is the number of healthy host cells, and $x_{3}$ is the number of effector immune cells. $a_{1,..,5}$ and $r_{1,2}$ are constants obtained by rescaling the original system model, which are described in [34], to obtain the dimensionless equations (5). These constants are related respectively to the loss of the tumor cell population, the tumor cell killing rate by the effector cells, the rate by which the tumor cells inactivate the healthy cells, the rate by which the tumor cells inactivate the effector cells, the death rate of the effector cells, the growth rate of the healthy tissue cells and the direct dependence of the stimulation of the immune system on the number of tumor cells. This model contains 7 multiplier operations and one division operation making it hardware demanding.

**FIGURE 4.**
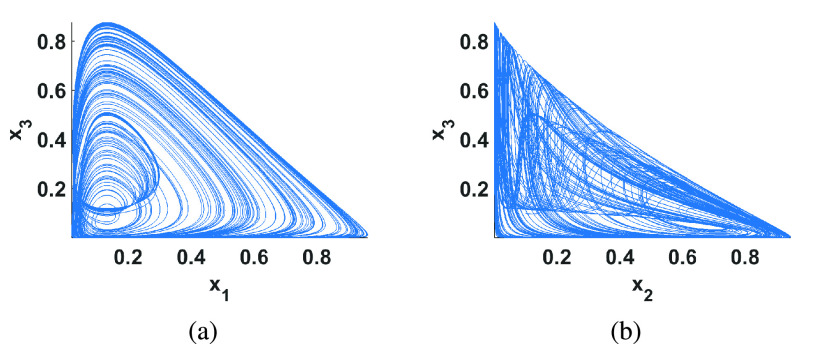
Numerical simulations of the discretized Cancer model with initial conditions $x_{1}(0)=x_{2}(0)=x_{3}(0)=0.1$ and $h=0.005$ (a) projection in the $x_{1}-x_{3}$ plane and (b) projection in the $x_{2}-x_{3}$ plane.

**FIGURE 5.**
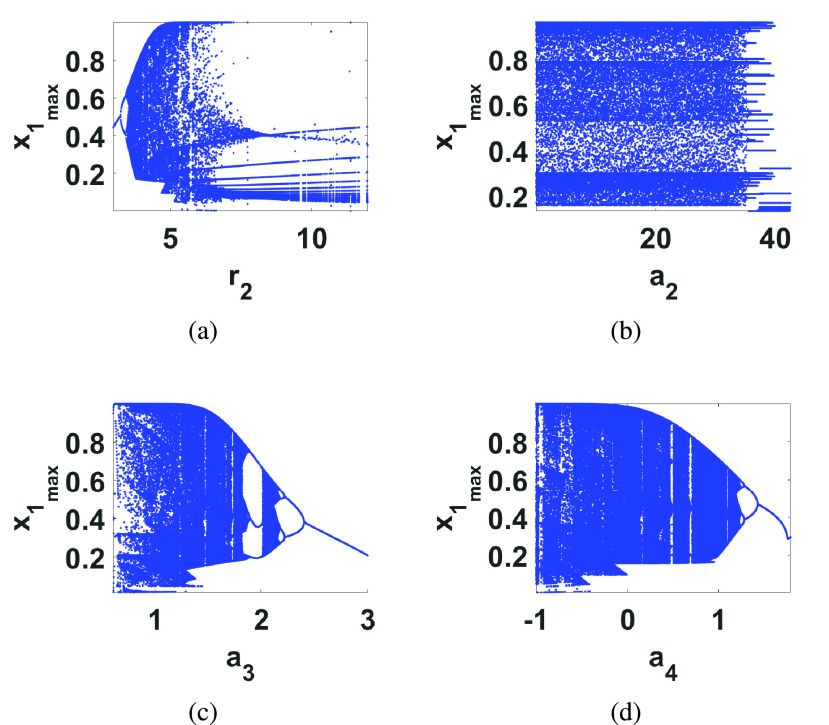
Bifurcation diagrams of the discretized Cancer model against (a) $r_{2}$, (b) $a_{2}$, (c) $a_{3}$ and (d) $a_{4}$ with the same initial conditions of Fig. 4.

Simulation results of this model after being discretized with an Euler method are shown in Fig. 4 with $(a_{1},a_{2},a_{3},a_{4},a_{5},r_{1},r_{2})=(1,2.5,1.5,0.2,0.5,0.6,4.5)$. For this system, the study of the effects of the parameter variations on the state variables using bifurcation diagrams revealed interestingly that it exhibits a period doubling route to chaos against $r_{2}$ and reverse bifurcation against both $a_{3}$ and $a_{4}$, as shown in Fig. 5.

## III. FPGA Implementations

### A. Using Altera Cyclone IV

**FIGURE 6.**
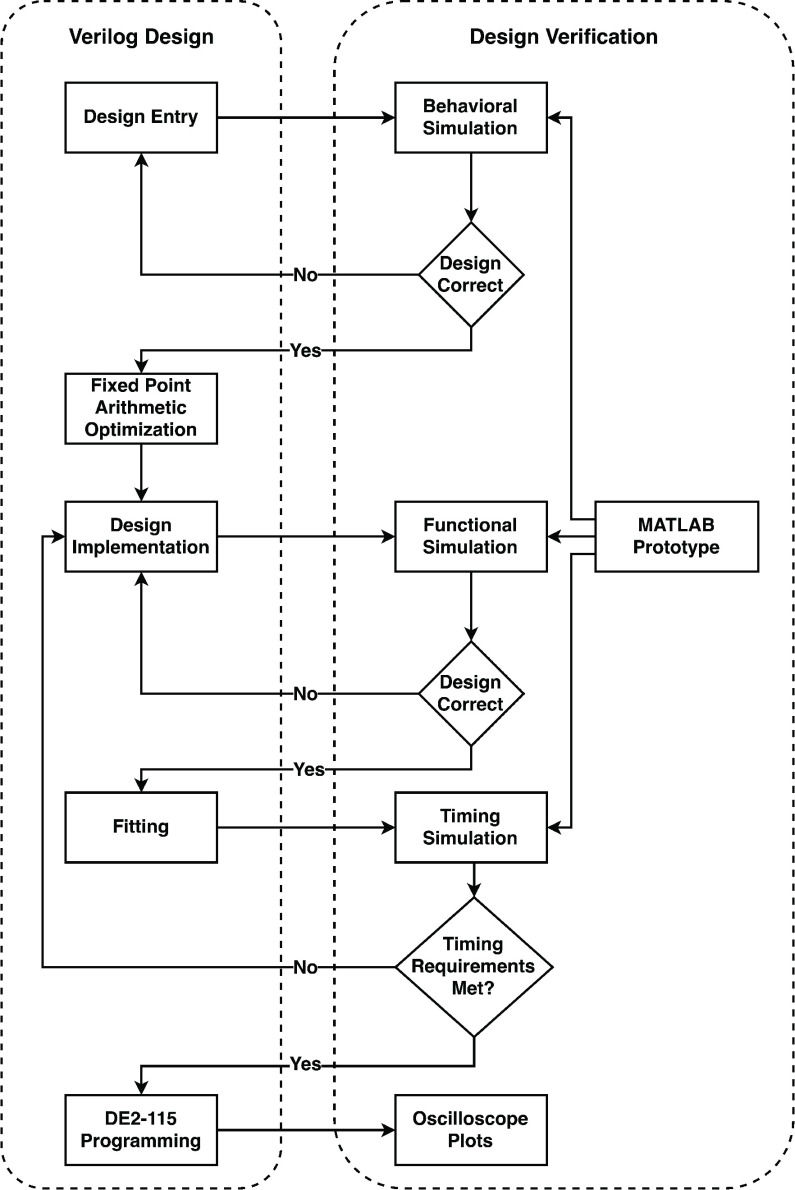
FPGA Design flowchart.

Figure 6 depicts the FPGA design process applied to implement three of the studied models (Ebola, Influenza and Cancer). This process consists of two parallel phases, namely the Verilog Design and the Design Verification phases. The two phases run in parallel to ensure that the hardware implementation meets all the specifications of the system at different key points during the process. The design steps allow for an iterative mechanism.

#### 1) Fixed-Point Architecture

**FIGURE 7.**
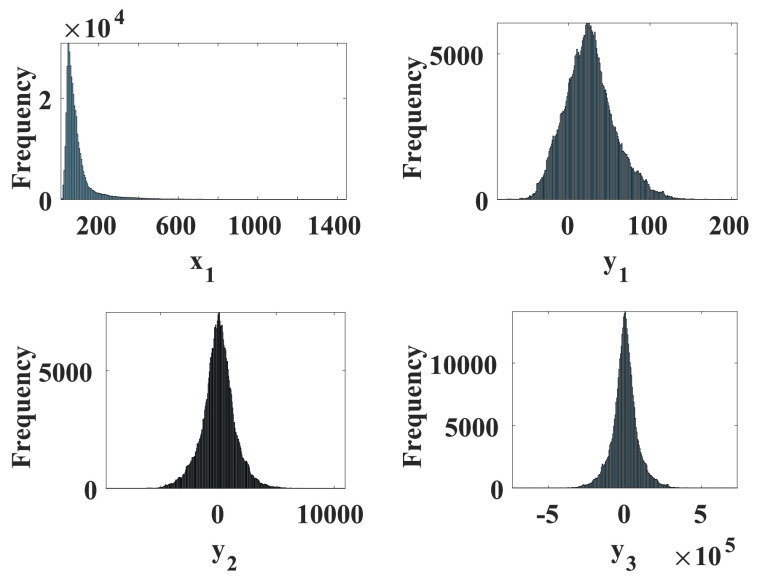
Frequency histograms showing the distribution of values in the four states of the Ebola model.

Floating-point units typically need more than one clock cycle to produce an output and consequently, synchronizing fixed-point results is much easier and usually produces a simpler and faster hardware design [35]. In fixed-point architectures, memory and bus widths are smaller, contributing significantly to a lower cost and power consumption [36]. That is why a fixed-point architecture is used to implement the hardware components of the three models. The fixed-point signed notation of $(1.m.n)$ is used to represent the number of bits allocated to the integer part $m$ and the fraction part $n$ of the number. The number of bits was determined by taking into account the maximum possible value of all state variables in the chaotic models. Both the Ebola and Cancer models use an internal architecture of 64-bit signed fixed-point whereas, the Influenza model requires more precision in the fractional part and hence a 256-bit signed fixed-point architecture is used. A typical histogram of the distribution of values for the four-state variables in the Ebola model is shown in Fig. 7. The more spread the values are, the less the required precision to discriminate among successive values.

#### 2) Hardware

**FIGURE 8.**
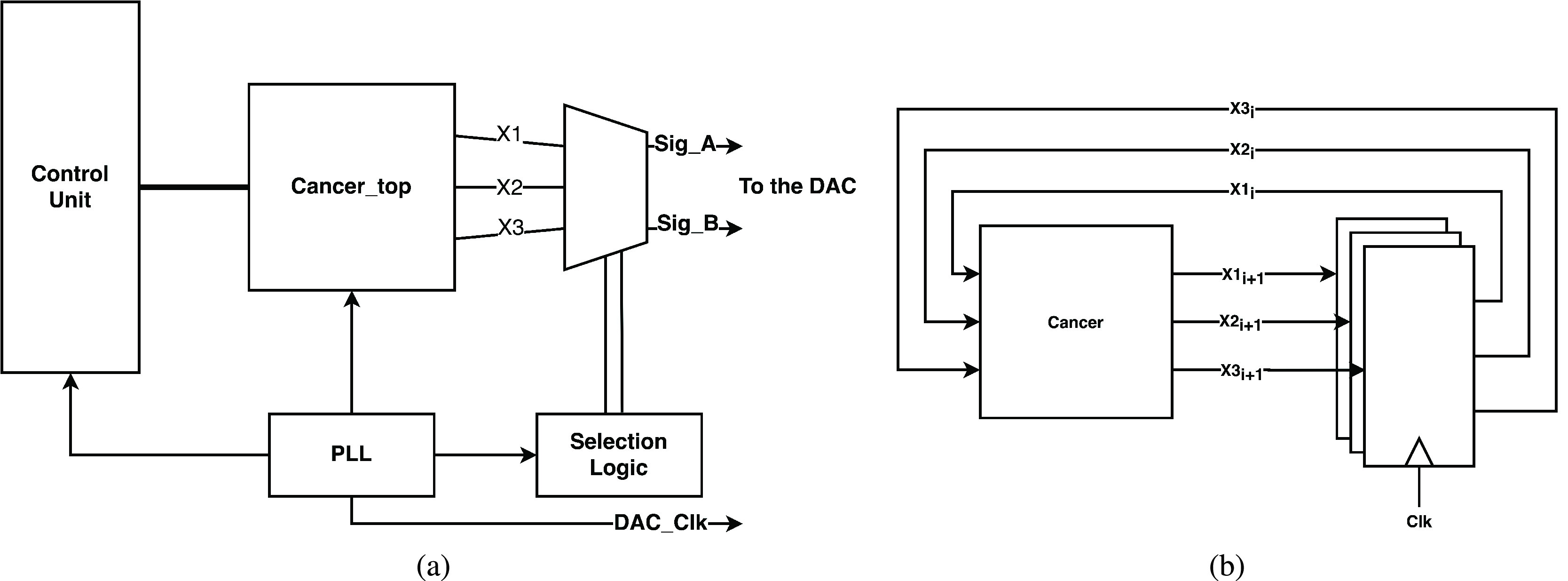
Architecture of (a) Cancer model top-level module and (b) the module *Cancer_top*.

Here the details of implementing the Cancer model (as an example) are discussed. This model is the most complex from an implementation perspective. Figure 8(a) shows the architecture of the top-level module of the Cancer model. One of the main components of this module is the phase-locked loop which is responsible for generating the clock frequency required to run the model blocks. This clock frequency is fed to the control unit which is responsible for controlling the number of iterations through a state machine. The *Cancer_top* module, shown in Fig. 8(b), is responsible for generating the values of the next state from the existing state values in the model. The selection logic enables the user to route any two-state variables to a digital-to-analog converter for external observation on an oscilloscope.

**FIGURE 9.**
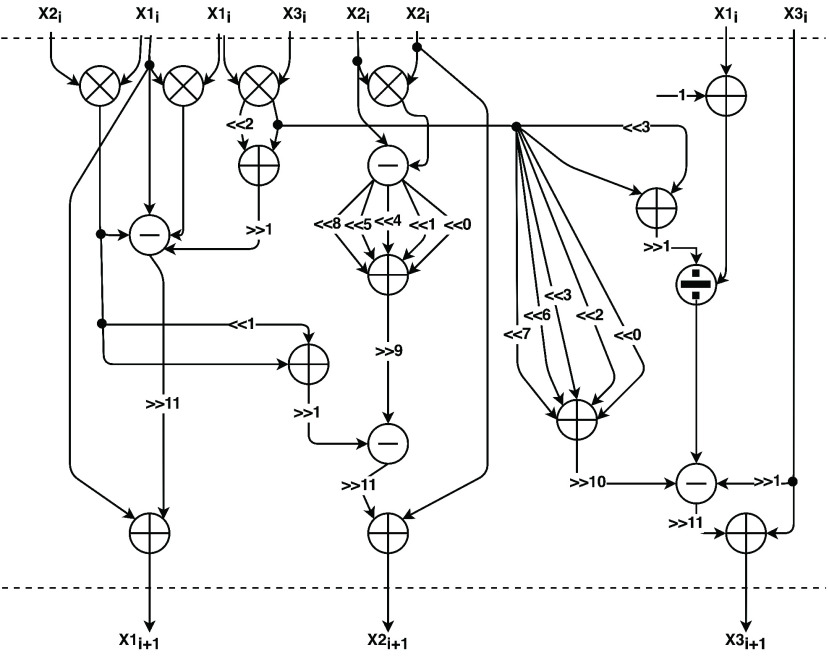
Internal architecture of the *Cancer* module.

Figure 9 shows the internal architecture of the combinational cancer module particularly the arithmetic and shift operations needed. The arithmetic operations are implemented using IP cores from Intel. To optimize the multiplication operation for example by a constant equal to 2.5, we first shift by 2 bits, which is a multiplication of 4, then we add the result to the original value resulting in a multiplication by 5. Finally, we shift the product 1 bit to the right in order to divide by 2. This enables us to compute a multiplication by 2.5 without the need to use the resource-consuming multiplication operation. The dotted lines in the figure are the state registers used to store the values of the three state variables of the model. These state registers enable synchronize the inputs and outputs with the clock cycles.

#### 3) Timing Analysis

Timing analysis is performed using the Timing Quest Timing Analyzer tool in Intel Quartus Prime software to find the maximum frequency at which the realized models can operate. The minimum input and output delays are set to 2ns, whereas the maximum input and output delays are set to 3ns. The maximum frequencies at which we can operate our three modules are found to be 17.02MHz, 10.86MHz, and 1.62MHz for the Ebola, Influenza, and Cancer models, respectively. The reduction in operating frequency in the Influenza model is mainly attributed to the necessary higher precision when compared to the Ebola model (256-bits vs. 64-bits). The drastic reduction in frequency in the Cancer model mainly results from the complex division operation needed to create $\dot {x}_{3}$. The PLL in all three designs was used to provide the clocks at maximum frequencies.

### B. Using Xilinx ARTIX-7

**FIGURE 10.**
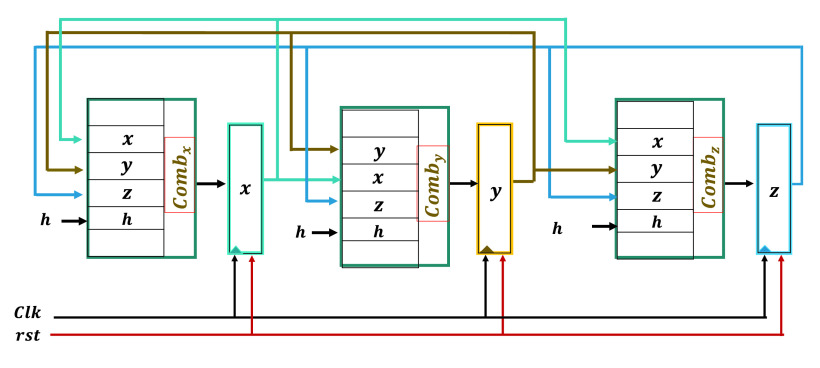
General block diagram for a 3D dynamical system, where a three combinational circuits are used to compute $x, y$ and $z$.

**FIGURE 11.**
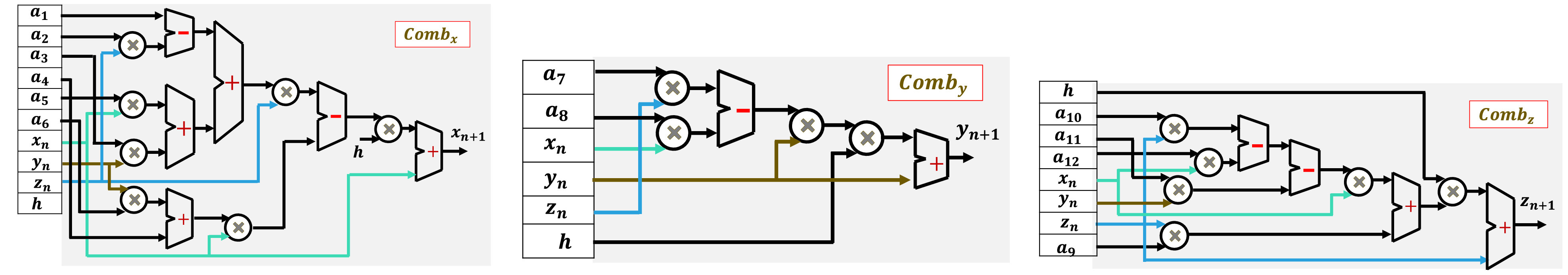
Combinational circuits block diagram for the numerical solution of $x, y$ and $z$ for the Covid 19 model.

**FIGURE 12.**
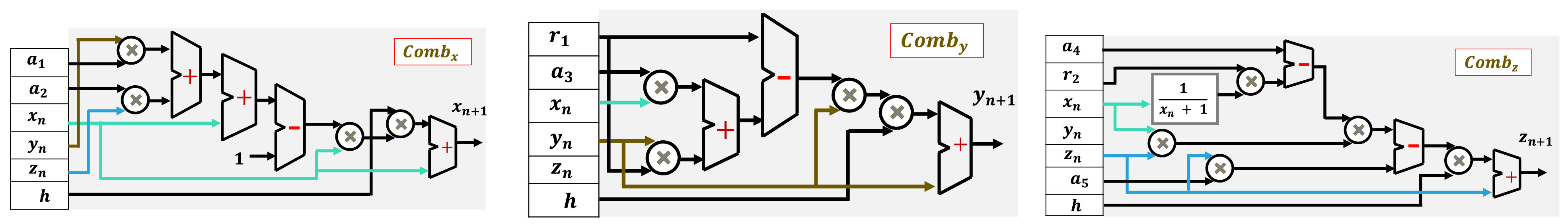
Combinational circuits block diagram for the numerical solution of $x, y$ and $z$ for the proposed cancer chaotic system.

Figure 10 shows the general block diagram used where a 3D chaotic system is considered with outputs $x, y$ and $z$ stored in three registers. Three combinational circuits are used to compute the numerical solution for $x, y$ and $z$. Taking the Covid 19 chaotic system as an example for this design procedure, Fig. 11 displays the three combinational circuits required to calculate the numerical solution of $x, y, z$ which correspond respectively to $C, S$, and $D$. Different arithmetic blocks are used to compute the numerical solution including adders, subtractors, and multipliers. 32-bit fixed points are used for the implementation each with 4-bits for the integer part and 28-bits for the fractional part. For the Cancer model, Fig. 12 presents the three combinational circuits needed to compute the numerical solution of $x, y$, and $z$ which represent $x_{1}, x_{2}$ and $x_{3}$ for this model respectively. The term $\frac {1}{x_{n}+1}$ is computed based on a linear approximation method. The linear binomial coefficients are generated based on MATLAB curve fitting. Since the range of $1+x$ is [1:2], the linear approximation is applied for an input interval [1:2]. Four uniform segments are used for the approximation. Here 64 bits fixed point are used each with 16-bits for the integer part and 48-bits for the fractional part. The outputs are truncated to 12-bits and hence the three registers $x, y$, and $z$ use 12 bits.

## IV. Results

**FIGURE 13.**
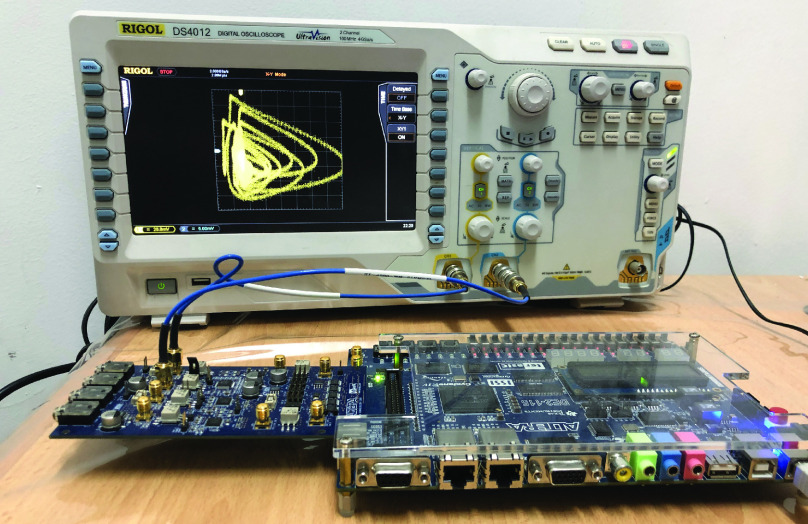
Experimental setup running the Ebola model on the Altera Cyclone IV FPGA board and showing the $x_{1}-y_{1}$ chaotic attractor.

The first implementation described in section III-A was based on the DE2-115 development board equipped with Altera Cyclone IV EP4CE115F29C7 FPGA device. This board includes many input/output (I/O) peripherals including switches and a High-Speed Mezzanine Card (HSMC) connector. A Texas Instruments high-speed Digital to Analog Converter (TI DAC5672) was connected to the HSMC connector on the DE2-115 board. The TI DAC5672 is a 14-bit dual-channel DAC, hence the main outputs of the three models needed to be scaled down to 14 bits in order to be displayed on the oscilloscope (RIGOL DS4012). Figure 13 shows the final experimental setup while the FPGA is running the Ebola model code. Table 1 summarizes the FPGA resource utilization for each of the three implemented models with this FPGA.

**TABLE 1:** FPGA Resources Utilization on Altera Cyclone IV

Resource	Ebola	Influenza	Cancer
Logic elements	4462/114480 (4%)	3874/114480 (4%)	5182/114480 (5%)
Registers	300	533	246
Embedded multiplier (9-bit)	96/532 (18%)	127/532 (24%)	48/532 (9%)
Pins	37/529 (7%)	34/529 (6%)	36/529 (7%)
PLL	1/4 (25%)	1/4 (25%)	1/4 (25%)
Frequency (MHz)	17	10.8	1.6

**FIGURE 14.**
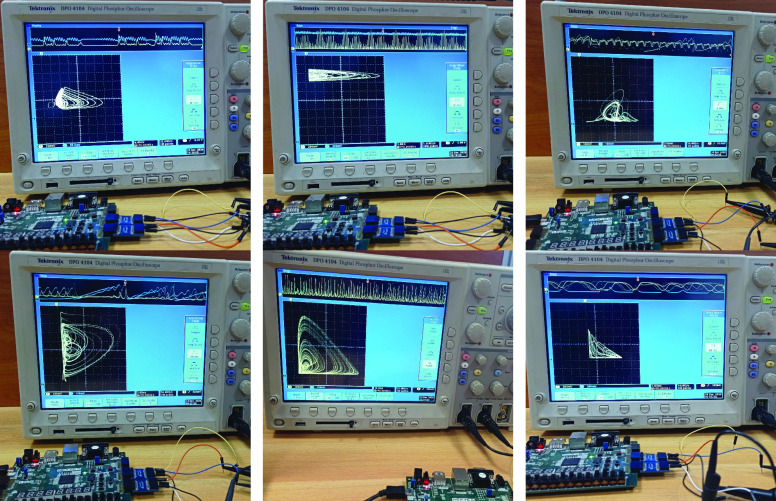
Experimental results using the Xilinx Artix-7 FPGA board and showing from left to right (in sequence) the chaotic attractor projection: $x_{1}-y_{1}$ of the Ebola model, $I-R$ of the Influenza model, $C-S$ of the Covid-19 model, $S-D$ of the Covid-19 model, $x_{1}-x_{3}$ of the Cancer model and $x_{2}-x_{3}$ of the Cancer model.

For the second implementation described in section III-B the Xilinx XC7A100TCSG324 FPGA was used. Table 2 presents a summary of the resources needed to implement the Covid-19 and Cancer models. The Covid 19 model is the most resource demanding of all models. Figure 14 shows the oscilloscope output from all epidemic models using this FPGA.

**TABLE 2:** Hardware Resources Summary for COVID 19 and Cancer Chaotic Systems Using a Xilinx XC7A100TCSG324 FPGA. The Throughput is Computed for 12 bits Output

Logic Utilization	Total No. Slices	Total No. Slices Registers	Maximum Frequency MHz	Throughput
Covid 19	605	288	23.263	0.279
Cancer	157	152	37.927	0.455

## V. Conclusion

We reported on the feasibility of implementing four complex chaotic models for virus spreading (Ebola, Influenza and Covid 19) and disease progress (Cancer) on two different FPGA platforms using fixed-point architectures. Although these models require a large number of multiplier blocks and have many parameters, their efficient realization on FPGAs is possible. Future work on applying and investigating the robustness of chaos control techniques to these epidemic models in real-time is ongoing.
